# Methodological Issues in Soccer Talent Identification Research

**DOI:** 10.1007/s40279-019-01113-w

**Published:** 2019-06-03

**Authors:** Tom L. G. Bergkamp, A. Susan M. Niessen, Ruud. J. R. den Hartigh, Wouter G. P. Frencken, Rob R. Meijer

**Affiliations:** 1 Department of Psychometrics and Statistics, Faculty of Behavioral and Social Sciences, University of Groningen, Grote Kruisstraat 2/1, 9712TS Groningen, The Netherlands; 2 Department of Developmental Psychology, Faculty of Behavioral and Social Sciences, University of Groningen, Grote Kruisstraat 2/1, 9712TS Groningen, The Netherlands; 3 Center for Human Movement Sciences, University of Groningen, University Medical Center Groningen, Hanzeplein 1, 9713 GZ Groningen, The Netherlands; 4 Football Club Groningen, Groningen, The Netherlands

## Abstract

Talent identification research in soccer comprises the prediction of elite soccer performance. While many studies in this field have aimed to empirically relate performance characteristics to subsequent soccer success, a critical evaluation of the methodology of these studies has mostly been absent in the literature. In this position paper, we discuss advantages and limitations of the design, validity, and utility of current soccer talent identification research. Specifically, we draw on principles from selection psychology that can contribute to best practices in the context of making selection decisions across domains. Based on an extensive search of the soccer literature, we identify four methodological issues from this framework that are relevant for talent identification research, i.e. (1) the operationalization of criterion variables (the performance to be predicted) as performance levels; (2) the focus on isolated performance indicators as predictors of soccer performance; (3) the effects of range restriction on the predictive validity of predictors used in talent identification; and (4) the effect of the base rate on the utility of talent identification procedures. Based on these four issues, we highlight opportunities and challenges for future soccer talent identification studies that may contribute to developing evidence-based selection procedures. We suggest for future research to consider the use of individual soccer criterion measures, to adopt representative, high-fidelity predictors of soccer performance, and to take restriction of range and the base rate into account.

## Key Points

**Table:** 

A broad selection of soccer talent identification studies are considered and their methodology, in terms of design, validity, and utility, is evaluated.
Four major methodological limitations are identified and discussed: the use of performance levels as the criterion; the focus on components as predictors of soccer performance; the influence of restriction of range on the generalization of findings; and the impact on the base rate on the utility of talent identification procedures.
To increase the robustness of its research practices, we propose that future soccer talent identification studies should adopt more individual soccer performance outcomes, high-fidelity predictors, where possible correct for range restriction, and take the base rate into account.

## Introduction

Sports organizations invest substantial resources in the search for players who have the potential to excel. These identification programs are aimed at detecting talented players who demonstrate strong performance in sport-specific abilities that are predictive of future career success [1–3]. Typically, these players are selected and recruited for specialized development programs that provide the appropriate learning conditions, facilities, equipment, and staff to realize the players’ potential [4, 5].

Historically, talent identification programs are associated with the subjective evaluation of players’ potential by coaches and scouts, who base their criteria primarily on personal taste, knowledge, and experience [6, 7]. However, in the last few decades, there has been an increasing interest in complementing these subjective assessments with evidence-based talent identification procedures, in order to increase the probability of selecting successful players. As a result, talent research has seen the integration of multidimensional and comprehensive models that detail prerequisites and predictors of successful adult performance [1, 8, 9], as well as a plethora of studies that have aimed to estimate the empirical relationships between these predictors and performance criteria in different sports.

Predicting future sports performance is inherently multifaceted and complex. Players’ developmental trajectories are rarely linear because cognitive and motor skills are intertwined and develop through dynamic interactions with the individual athlete’s performance environment [10–14]. Several recently published systematic reviews have aimed to summarize the empirical evidence for factors that may determine elite sports performance in general [15, 16], and in specific domains such as soccer [17–19]. Results from these studies indicate that various physical, technical, tactical, and psychological factors contribute to determining individual sport-specific success. However, due to the considerable variation in study designs, findings across individual talent identification studies are inconsistent and difficult to compare [15, 18, 20, 21], and therefore there is no clear set of variables that uniformly predict skill level [15, 22].

Still, a major aim in the field of sport sciences is to apply best-practice talent identification methods, that is, methods that allow for valid predictions of players’ future performance. To date, various articles have been published discussing scientific or ethical challenges that hinder the possibilities of identifying talents [16, 22–24], such as the definition of the concept of talent [24], the influence of maturation on performance [7], and the difficulties of early selection and early prediction of adult performance based on knowledge of how (physical) performance characteristics develop [2, 13, 25, 26]. Furthermore, several papers have discussed methodological and design features of talent identification studies [18, 19, 22]. However, we observed that reflections of methodological issues specifically relevant for research on predictors and criteria used for selection purposes are scarce in the talent identification literature. Critical reflections on these issues are important for providing insight into how research results should be interpreted, and to provide guidelines for researchers in employing best practices from a methodological point of view.

The aim of this position paper is to provide an overview of the talent identification literature and discuss some methodological issues that we consider particularly relevant in the context of selection. More specifically, we discuss methodological considerations commonly addressed in psychological research on selection (further referred to as selection psychology) regarding determinants of predictive validity, utility, and interpretability of assessment and selection procedures. Selection psychology is concerned with how to best select candidates for different achievement domains [12, 27, 28]. It provides psychometric and statistical tools for measuring human traits, skills, abilities, and performance, and defines theoretical principles that affect the relationship between a (set of) predictor(s) and a criterion. While research in selection psychology has mostly focused on selecting candidates for jobs, its psychometric and statistical considerations are relevant for a wide range of performance and expertise contexts that involve selection, including higher education [12, 29, 30] and sports [12, 31].

Based on the selection psychology framework, we discuss four methodological topics that are relevant for talent identification research in soccer.^1^ Furthermore, we offer suggestions based on these topics that can improve the design of future talent identification studies and can contribute to the development of evidence-based talent identification practices. These topics are (1) the operationalization of criterion variables (the performance to be predicted); (2) the fidelity of the performance indicators used as predictors; (3) the effects of range restriction on the predictive validity of predictors used in talent identification; and (4) the effect of the base rate on the utility of talent identification procedures. Some of these issues have been briefly touched upon previously in the context of talent identification in sports [8, 22, 24, 32], but they are rarely thoroughly addressed (for an exception on some issues, see Ackerman [33]). Moreover, since these issues are not explicitly and specifically accounted for, we consider an in-depth evaluation valuable for advancing the field.

Because the aim of this article is to relate some specific methodological principles that are relevant in research on selection, and thereby for talent identification in soccer, we do not discuss analytic and design-related issues that have been discussed previously. Examples are the use of stepwise model selection methods [34, 35], presenting exploratory results as confirmatory findings [36, 37], the absence of cross-validation, issues related to multiple testing [38], and the use of small sample sizes, which are issues that are relevant across various scientific disciplines.

## Methodological Issues

### Operationalizing the Criterion

Talent identification in soccer involves the measurement of skills and abilities [1, 2, 22] that are related to an indicator of soccer performance (the criterion). This criterion is ideally measured in the future (predictive validity), but is sometimes measured at the same time (concurrent validity). In our view, the talent identification literature has largely neglected to pay attention to the operationalization of criterion variables that provide information about the differences between players in terms of soccer performance after selection [39]. More specifically, an explicit measure of soccer performance is rarely used as a criterion. Instead, the criterion used in most studies is the selection decision itself, which is usually a categorical variable indicating performance or skill level. Examples of performance-level indicators that have been used in studies are elite, sub-elite, and non-elite level [40–42]; professional, semi-professional, or non-professional level [43–45]; first team or reserves [46]; elite, club level, or dropouts [47, 48]; national or regional level [49–51]; selected and non-selected players [52–55]; and nationally drafted or non-drafted players [56] (see Table 1).

**Table 1:** Design and methodological characteristics of soccer talent identification studies

Study	Prognostic period (follow-up)	Age at assessment	*N*	Criterion	Predictors	Considers restriction of range
Reilly et al. 2000 [3]	Cross-sectional	U17	16 15	Elite Sub-elite	*Low-fidelity:* Height, weight, body composition (*physical*—7 variables) Speed, endurance, agility, strength (*physiological*—10 variables) Dribbling and shooting (*soccer-specific*—2 variables) Anxiety intention and direction, anticipation, motivation (*psychological*—11 variables)	Partially—authors briefly consider if findings will replicate in highly selected players who are exposed to more systematic training
Vaeyens et al. 2006 [73]	Cross-sectional	U13–U16	490^a^	Elite Sub-elite Non-elite	*Low-fidelity:* Height, weight (*physical*—3 variables) Speed, endurance, agility, strength (*physiological*—10 variables) Dribbling, shooting, passing, juggling (*soccer-specific*—4 variables)	Yes—authors consider that differentiating the ability of performance indicators might be dependent on competitive age class, and relate findings to homogeneity of sample due to preselection
Toering et al. 2009 [75]	Cross-sectional	U12–U18	159 285	Elite Non-elite	*Low-fidelity:* Self-regulation (*psychological*—6 variables)	No, but authors did control for effects of age
Coelho e Silva et al. 2010 [84]	Cross-sectional	U14	69 45	Elite Local	*Low-fidelity:* Maturity (3 variables) Height, weight, body composition (*physical*—3 variables) Speed, endurance, agility, and power (*physiological*—5 variables) Dribbling, shooting, passing (4 variables) Task and ego orientation (*psychological*—2 variables) *Other:* Soccer experience (1 variable)	No
Waldron and Worsfold 2010 [40]	Cross-sectional	U14	69 32	Elite Sub-elite	*High-fidelity:* Attempted, successful and unsuccessful skill involvements in a match, such as passing, shooting, tackling (18 variables)	No
Kavussanu et al. 2011 [42]	Cross-sectional	U13–U17	69 49	Elite Non-elite	*Low-fidelity:* Task and ego orientation, perceived parental environment (*psychological*—11 variables)	No
Waldron and Murphy 2013 [100]	Cross-sectional	U15	15 16	Elite Sub-elite	*Low-fidelity:* Speed, strength, agility (*physiological*—5 variables) Dribbling (*soccer-specific*—2 variables) *High-fidelity:* Attempted, successful and unsuccessful skill involvements in a match, such as passing, shooting, tackling (6 variables) Physiological performance during games, such as intensity movements and distance covered (9 variables) *Other:* Heart rate and perceived exertion (2 variables)	No
Haugaasen et al. 2014 [44]	Cross-sectional	U14–U22	615 81	Non-professional Professional	*Other:* Engagement in soccer-specific activities (sociological—4 variables)	Partially—authors specifically examine participation in soccer-specific activities in different age categories, but do not relate their findings to the homogeneity of the sample, due to preselection
Verburgh et al. 2014 [77]	Cross-sectional	U9–U17	84 42	Highly-talented Amateur	*Low-fidelity:* Executive functions (*psychological*—8 variables)	Partially—authors briefly state that findings can only be considered in the context of the samples, but authors do not examine the differentiating ability of predictors per age category, and did not control for age
Baláková et al. 2015 [79]	Cross-sectional	U14	91^a^	Talented Less-talented	*Low-fidelity:* Cognitive functions (*psychological*—16 variables)	No
Goto et al. 2015 [54]	Cross-sectional	U9–U10	14 20	Retained Released	*Low-fidelity:* Maturity (1 variable) *High-fidelity:* Physiological performance during games, such as intensity movements and distance covered (6 variables)	No
Huijgen et al. 2015 [41]	Cross-sectional	U14–U18	47 41	Elite Sub-elite	*Low-fidelity:* Lower and higher cognitive functions (*psychological*—*6 variables)*	No
Fenner et al. 2016 [69]	Cross-sectional	U10	16	Rating of technical performance in SSG^b^	*Low-fidelity:* Speed, strength (physiological—3 variables) *High-fidelity:* Individual performance in SSGs, time–motion characteristics (5 variables)	Yes—authors compare findings to a similar study with older players, and suggest that these findings did not replicate due to the increased homogeneity of technical skills in the older players.
Bennett et al. 2017 [101]	Cross-sectional	U12–U16	36 37	High-level Low-level	*High-fidelity:* Attempted, successful and unsuccessful skill involvements in a match, such as passing, shooting, dribbling (13 variables)	No
Den Hartigh et al. 2017 [55]	Cross-sectional	U11	49 39	Selected Non-selected	*Low-fidelity:* Game reading based on video images (1 variable)	No
Rowat et al. 2017 [71]	Cross-sectional	U18	27	Technical performance in SSG rating^b^	*Low-fidelity:* Maturity (1 variable) Speed, endurance (*physiological*—2 variables) Dribbling, passing, shooting (*soccer-specific*—4 variables)	No
Wilson et al. 2017 [39]	Cross-sectional	NA	32	Individual performance in 1-vs-1 and 11-a-side games^b^	*Low-fidelity:* Height, weight, body composition (*physical*—7 variables, 2 latent variables) Speed, strength, balance (*physiological*—7 variables, 3 latent variables) Dribbling, juggling, shooting, passing (*soccer-specific*—5 variables, 2 latent variables)	No
Gil et al. 2007 [107]	< 1 year	U15–U18	126 68	Selected Non-selected	*Low-fidelity:* Height, weight, body composition (*physical*—22 variables) Speed, endurance, agility, power (*physiological*—10 variables)	Partially—authors briefly consider that technical, tactical and psychological skills may have more discriminative power for selected players at later ages, when growth differences are less important
Gravina et al. 2008 [46]	< 1 year	U11–U14	44 22	First team Reserves	*Low-fidelity:* Height, weight, body composition (*physical*—13 variables) Speed, strength (*physiological*—10 variables)	Partially—authors very briefly relate findings to extended population, but do not discuss homogeneity of the sample due to preselection
Huijgen et al. 2014 [52]	< 1 year	U17–U19	76 47	Selected Deselected	*Low-fidelity:* Speed, endurance (*physiological*—4 variables) Dribbling (*soccer-specific*—4 variables) Tactical characteristic questionnaire (4—variables) Task and ego orientation, anxiety, concentration, motivation (*psychological*—8 variables)	No, but authors did control for effects of age
Lago-Penas et al. 2014 [63]	< 1 year	U15/U17/U20	156^a^	Selected Non-selected	*Low-fidelity:* Height, weight, body composition (*physical*—6 variables) Speed, endurance, strength (physiological—3 variables)	No
Zuber and Conzelmann 2014 [70]	< 1 year	U13	140	Overall soccer performance rating^b^	*Low-fidelity:* Achievement motive (*psychological*—2 latent variables) Speed, endurance, strength, agility (*physiological*—4 variables, 1 latent variable) Dribbling, juggling and ball control (*soccer-specific*—3 variables, 1 latent variable)	Yes—authors relate findings to homogeneity of the sample due to preselection
Aquino et al. 2017 [57]	< 1 year	U17	28 38	Selected Non-selected	*Low-fidelity:* Maturity (1 variable) Height, body composition (*physical*—3 variables) Speed, endurance, strength (*physiological*—7 variables) Shooting, ball control, dribbling, tactical skills questionnaire (*soccer-specific*—4 variables)	No
Gil et al. 2014 [53]	1 year	U10–U11	21 43	Selected Non-selected	*Low-fidelity:* Maturity (3 variables) Height, weight, body composition (*physical*—9 variables) Speed, endurance, strength (*physiological*—7 variables) *Other:* Soccer experience (1 variable)	No
Vestberg et al. 2012 [78]	< 2 years	Adult	29 28	High division Low division Goals scored and assists^b^	*Low-fidelity:* Executive functions (psychological—3)	Yes—authors also have results for non-soccer players, and are therefore able to compare results with the general population
Vestberg et al. 2017 [80]	< 2 years	U13–U20	30	Goals scored and assists^b^	*Low-fidelity:* Executive functions (*psychological*—4 variables)	Yes—authors also have results for non-soccer players, and are therefore able to compare results with the general population
Figueiredo et al. 2009 [47]	2 years	U12–U15	36 90 33	Drop-out Club Elite	*Low-fidelity:* Height, weight, body composition (*physical*—6 variables) Speed, endurance, agility, and power (*physiological*—6 variables) Dribbling, shooting, passing (*soccer-specific*—4 variables) Task and ego orientation (*psychological*—2 variables) *Other:* Soccer experience (1 variable) Rating of player’s potential (1—variable)	No
Deprez et al. 2015 [48]	2 years	U10–U17	633 231 29 29	Club Drop-out Contract No contract Total minutes played in first team^b^	*Low-fidelity:* Maturity (2 variables) Height, weight, body composition (*physical*—3 variables) Speed, power, endurance, motor coordination (*physiological*—8 variables) Dribbling (*soccer-specific*—2 variables)	Yes—authors examine the discriminatory power of variables per age group and discuss these results in relation to the homogeneity of each age group, in terms of physical abilities. They also briefly relate their findings to the extended, unselected population
Zuber et al. 2015 [50]	2 years	U13	10 82	National team Elite—not selected	*Low-fidelity:* Achievement motivation, achievement goal orientation, self-determination (psychological—5 variables)	Yes—authors investigate distinct clusters formed of the different variables, for each age category. They also briefly consider homogeneity of the sample on examined variables
Zuber et al. 2016 [49]	3 years	U12	12 39 68	National Regional No talent card	*Low-fidelity:* Maturity (1 variable) Net hope (*psychological*—2 variables) Speed, endurance, strength (*physiological*—3 variables) Dribbling, passing, juggling (*soccer-specific*—3 variables)	Yes—authors investigate distinct clusters formed of the different variables, for each age category. They also note that results should only be considered in the context of their homogenous sample, and cannot directly be translated to the general population
Zibung et al. 2016 [51]	3 years	U13	10 30 64	National talent card Regional talent card No talent card	*Low-fidelity:* Speed, endurance, agility (*physiological*—3 variables) Dribbling, passing, juggling (*soccer-specific*—3 variables)	Yes—authors briefly discuss the decrease of variance in performance over time, as a result of increasing homogeneity of the sample due to preselection
Huijgen et al. 2013 [82]	1–3 years	U12–U19	269 50	Selected De-selected	*Low-fidelity:* Passing: Loughborough Soccer Passing Test (*soccer-specific*—2 variables)	Partially—authors take the development of skills into account and relate the results to different age categories, but only very briefly consider homogeneity of the sample due to preselection
Höner and Feichtinger 2016 [21]	4 years	U12	308 2369	Youth academy No youth academy	*Low-fidelity:* Achievement motive, ego orientation, sport orientation, volition, self-concept, self-efficacy, anxiety (*psychological*—17 variables)	Yes—authors relate their findings to the homogeneity of the sample due to preselection
Kannekens et al. 2011 [83]	3—5 years	U17—U19	52 53	Professional Amateur	*Low-fidelity:* Tactical skills questionnaire (soccer-specific—4 variables) *Other:* Soccer experience, practice per week, non-specific sport practice	No
Gonaus and Müller 2012 [56]	1–6 years	U14–U17	821 3912	Drafted Non-drafted	*Low-fidelity:* Speed, endurance, strength, agility (*physiological*—12 variables)	Yes—authors consider the homogeneity of the sample and relate the discriminating power of variables to a specific age group
le Gall et al. 2010 [64]	4–6 years	U14–U16	48 167 235	International Professional Amateur	*Low-fidelity:* Maturity (3 variables) Height, weight, body composition (*physical*—3 variables) Speed, endurance, agility, and power (*physiological*—14 variables)	Partially—authors examine the discriminative power of performance characteristics per age group, but only very briefly consider how homogeneity of their sample due to preselection may affect findings
Höner and Votteler 2016 [43]	4–7 years	U12	195 731 1025 20,892	National Regional Academy Not selected	*Low-fidelity:* Sprinting, agility (*physiological*—2 variables) Dribbling, ball control, shooting (*soccer-specific*—3 variables)	Yes—authors mention restriction of range, relate findings to homogeneity of the sample due to preselection, and consider that discriminatory power may vary according to age group and homogeneity of the sample
Höner et al. 2017 [45]	8–10 years	U12	89 913 13,176	Professional Semi-professional Non-professional	*Low-fidelity:* Relative age (1 variable) Height, weight (*physical*—2 variables) Speed, agility (*physiological*—2 variables) Dribbling, shooting, ball control (*soccer-specific*—3 variables)	Partially—authors briefly consider how predictive value may differ for different age categories, but do not discuss homogeneity of their sample due to preselection
Van Yperen 2009 [76]	15 years	U15–U18	18 47	Successful Unsuccessful	*Low-fidelity:* Goal commitment, coping, social support (*psychological*—3 variables) *Other:* Assessment of initial performance by coaches (1 variable)	No, but the author did control for initial performance level
Martinez-Santos et al. 2016 [74]	2–18 years	Adult	74 161	First/second division Semi-professional	*Low-fidelity:* Speed, strength (*physiological*—3 variables)	No

Electronic databases (MEDLINE, SPORTDiscus, Google Scholar) were searched between 2000 and 2018 for empirical studies on talent identification, using the following combination of terms: talent identification OR selection OR prediction and performance and soccer OR football. Additionally, snowballing was used to identify other relevant studies. Studies were included if they met the following criteria: (1) focused on soccer or association football; (2) aimed to relate empirically multidimensional abilities and skills (e.g. physical, physiological, psychological, technical, tactical) or assessment methods to soccer performance or skill level; and (3) were peer-reviewed journal articles written in English. To restrict our sample, we excluded studies that focused predominantly on other types of football (e.g. futsal, American Football, Australian Rules football), and goalkeepers. Moreover, we excluded studies that mainly focused on the effects of relative age, maturity and genetic disposition. Although these topics are highly relevant for understanding talent development, we believe they warrant their own discussion and are therefore not within the scope of this paper. Finally, both cross-sectional and longitudinal studies were included. Although the empirical value of cross-sectional studies is limited compared with those with longitudinal designs, the methodological topics that are addressed in this paper also apply to those studies

*U* Under, i.e. U18 means under the age of 18 years, *SSG* small-sided game*, NA* not available

^a^The exact number of players per performance level could not be retrieved

^b^An individual soccer criterion measure, instead of performance or skill level

The operationalization of soccer performance as performance level is appropriate if a talent researcher wants to understand factors that distinguish players *perceived* as talented from those perceived as ‘less talented’ [52, 57]. Furthermore, the use of performance level as a criterion measure makes sense from a practical perspective because measuring individual soccer performance objectively is difficult [58]. In contrast to individual sports such as track and field and swimming, there is no definite measure of an individual’s performance in an open-skilled sport such as soccer [3]. Therefore, researchers may use performance level as a practical instrument that is expected to represent an indirect measure of the players’ general soccer performance as assessed by coaches and scouts, who typically evaluate players over an extended time period and take multidisciplinary performance factors into account [6, 59].

While using performance level as a criterion measure is understandable from a pragmatic point of view, it also carries some problems. First, this approach provides limited information on the individual differences between players [60, 61] on the actual outcome of interest, i.e. soccer performance in 11-a-side games [9]. We believe that the ultimate aim of soccer talent identification research is to predict individual *soccer performance* as a function of performance in talent identification procedures, not *selection* as a function of performance in talent identification procedures [39, 62]. Thus, talent identification procedures should strive to predict how players will perform, relative to others, but research designs that adopt a performance-level criterion implicitly assume that all players within a performance level perform equally well. As a result of this operationalization, the predictive value of talent predictors is often investigated using statistical analyses based on mean differences between the selected and non-selected players (mostly through the use of t-tests or [multivariate] analysis of variance; see Figueiredo et al. [47], Lago-Penas et al. [63], and le Gall et al. [64]). Although these statistical analyses can contribute to discovering relevant predictors for talent identification research to some extent, these designs cannot determine the value of different combinations of performance factors in predicting an outcome variable indicative of individual soccer ability [22, 39, 43].

Second, determining factors that predict individual soccer performance allows for successful selection of players on the basis of those variables. However, the use of a selection decision as the criterion can hinder this aim because the judgment of a player’s performance level might not be an accurate representation of individual soccer performance. This approach strongly depends on the validity of the coach’s or scout’s judgment in distinguishing between successful and ‘non-successful’ players. Yet, the validity of these judgments is not well-established, and is often even biased [12]. For example, judges are easily influenced by factors unrelated to a player’s talent or performance, such as the player’s skin color or reputation [65, 66]. In addition, the bias of judges to systematically select more mature players or players born earlier in the year has been well-reported in the talent identification literature [67, 68]. Thus, it is not clear whether predictors of perceptions of successful performance are also valid predictors of individual match performance after selection [24].

There are only a few studies within the talent identification literature that used individual soccer performance as an outcome measure. Examples include structured ratings of in-game performance [69–71], and metrics based on successful and unsuccessful skill involvements during games [39, 72]. As we discuss in Sect. 3.1, we believe that the validity and reliability of such measures requires closer examination in future research. Taken together, we argue that the criterion measures that are currently used in most talent identification studies are intuitive and straightforward, but have their shortcomings and are insufficiently validated for studies that aim to identify and understand what factors predict individual soccer performance. In contrast, a reliable and objective soccer-specific criterion measure is complicated to operationalize, but allows for the measurement of individual performance differences, so that the predictive value of different measures can be determined more meaningfully.

### Predictors of Soccer Performance

The predictors that have been studied in soccer talent identification research are strongly influenced by the classification scheme proposed by Williams and Reilly [1, 3], who classified predictors of individual soccer performance into four sport science disciplines: physical, physiological, psychological, and sociological. Examples of predictors include height, weight, and body composition (physical) [47, 53, 73]; speed, strength and endurance (physiological) [43, 52, 56, 74]; self-regulation, motivation, task and ego orientation, and cognitive functions (psychological) [3, 21, 50, 52, 75–80]; and hours of practice and perceived social support (sociological) [44, 76]. Other predictors that are derived from this classification scheme are technical skills, such as dribbling and passing technique, and self-assessed tactical skills [3, 45, 48, 81–84] (see Table 1).

Given the multifaceted nature of soccer performance, it makes sense to investigate the extent to which these variables combined predict success and individual performance. Different studies have demonstrated that some of these skills and abilities are able to discriminate between players of varying performance levels [15–18]. More importantly, the major advantage of this approach in talent identification procedures is that skills and abilities, such as intermittent endurance capacity, dribbling technique, and passing ability, are relatively straightforward to measure in a standardized and reliable way [85–87].

Although many studies have examined the predictive relevance of these variables in soccer, the reported effect sizes are generally small to moderate [18, 43, 45, 56]. An explanation from selection psychology for the limited predictive validities in soccer talent identification research may be related to the ‘fidelity’ of the predictors, that is, the extent to which the performance task mimics the criterion behavior in content and context. On one side of the fidelity continuum are low fidelity predictors, which have relatively little overlap with the criterion in terms of the behavior the player should show and the context in which the player must perform [31, 88]. These low fidelity predictors measure distinct, general performance components that are thought to be related to the criterion behavior. Such low fidelity predictors are referred to as ‘signs’ in the selection psychology literature [89]. Thus, most of the predictors classified by Williams and Reilly [1], such as height, speed, and motivation, can be characterized as signs because they measure distinct characteristics and lack fidelity to the criterion of soccer performance in terms of the task and or the context in which they are assessed [31].

The selection psychology literature shows that the predictive validity of assessment procedures often improves when the degree of fidelity increases, that is, when the predictor becomes more similar to the criterion in terms of behavior, task, and contextual constraints [8, 12, 90]. The underlying rationale is the notion of behavioral consistency: ‘the best predictor of future behavior is similar past or current behavior’ [89, 91–93]. Tests that assess soccer-specific technical skills, such as dribbling and passing technique, possess higher fidelity to the criterion of soccer performance than variables such as height, speed, and motivation. Accordingly, there is evidence that these predictors have better prognostic relevance [45, 82], and discriminate more consistently between skill groups than the latter group of variables [19, 39, 45]. Still, these tests measure distinct skills, and do not incorporate many of the necessary contextual constraints of in-game soccer performance, such as the task of scoring goals and the presence of moving opponents. In other words, such tests may still not mimic the criterion of interest, which is in-game soccer performance, to a large enough extent [60]. For example, the Loughborough Soccer Passing Test, a test frequently used to assess the passing ability of soccer players [82, 85], was recently found to be a poor predictor of passing performance during a match [94].

An important avenue therefore is to develop predictors that further minimize the ‘inferential leap’ from the predictor to the criterion, and thus possess even higher fidelity. One approach to establish such predictors in soccer is to take a ‘sample’ of the criterion performance in a highly representative context [31, 88], for example, in small-sided games (SSGs). SSGs are games played on reduced pitch areas and with fewer players (e.g. 4 vs. 4, or 7 vs. 7) than in an official match. Individual performance in SSGs can be considered a sample-based predictor because it is obtained based on behavior, task, and contextual constraints similar to those present in the criterion performance.

An important conclusion from the selection psychology literature is that sample-based assessments can be very good predictors of future performance [95–98], especially in homogeneous samples and for multidimensional outcome measures [99]. Because soccer talent identification research is often based on homogenous samples (e.g. players who are already in a talent program), and soccer performance is multidimensional [1], a samples approach to prediction is expected to result in greater predictive value [12]. Accordingly, several recent studies have related performance or skill level to predictors that we would characterize as sample-based, such as attempted and completed skill involvements (i.e. event data) within SSGs or regular games [40, 100, 101]. These sample-based predictors were relatively successful in distinguishing between groups of elite and sub-elite or non-elite players, and these results demonstrate how high-fidelity methods may be useful as alternatives to isolated components in predicting soccer performance [40, 100, 101]. However, similar to individual soccer performance criterion measures, the reliability of individual performance assessed through SSGs needs to be addressed in future studies (see Sect. 3.2).

Finally, the suggestion of samples as predictors of performance is also directly in accordance with theoretical developments in the field of motor learning and talent development regarding the use of representative designs for learning and assessment purposes [12, 102–104]. Several authors have already suggested that talent identification procedures should include more representative measures [8, 9, 15, 22]. In using samples as predictors of soccer performance, the interaction between different performance components is embedded in behavior that is representative of the criterion performance, thereby closing the gap between predictor and criterion.

In conclusion, soccer talent identification research has generally focused on low- or moderate-fidelity predictors of soccer performance, which has not only resulted in some interesting findings but also in an inconsistent body of evidence that does not provide clear guidelines for stakeholders in practice. The selection psychology literature suggests that high-fidelity measures may enhance the predictive value of talent identification procedures, but such methods are not often applied in the soccer talent identification literature yet.

### Restriction of Range

Talent identification studies often compare samples that are already highly restricted in terms of talent or skill, such as elite versus sub-elite athletes. In such cases, empirical relationships between performance indicators used as predictors and the criterion performance often deviate from relationships in the population [33]. This is a problem when, due to selection, a relatively homogenous sample that is not representative of the population of interest (containing all candidates, selected and not selected) is used to establish predictor–criterion relations [24]. As a result, predictor–criterion relationships obtained from such samples are usually underestimated because of ‘restriction in range’ [105].

To illustrate the effect of range restriction, we consider the study by le Gall et al. [64]. They examined anthropometric and physical characteristics of highly trained U14–U16 soccer players in a national academy, who, upon leaving the academy, achieved either international or professional status, or remained amateurs. The authors investigated the mean differences for 17 dependent variables, ranging from height, weight, and maturity measurements, to sprint and endurance performance and lower body explosiveness. Although statistically significant mean differences were found for some variables, there were no large differences between the groups on most performance indicators within age categories. For instance, in the U16 category, maximal anaerobic power and height distinguished between future internationals and amateurs with moderate effect sizes, but there was no strong evidence for vertical jump, 10-, 20-, 30-, and 40-meter sprint, and lower body explosiveness distinguishing between any combination of international, professional, and amateur players.

Based on these findings, the conclusion may be that these variables are not very useful for differentiating future career success in elite-level U16 players. However, it would be false to conclude that these characteristics are not important for attaining soccer-specific success *in general* [33]. It is likely that the sample of academy players were exposed to the same training routine, had similar practice histories, and were (directly or indirectly) preselected on at least some of the variables in this study. This preselection in an homogenous group of athletes in terms of physical performance results in a reduction in variance in the predictors and in the criterion. If the same predictors were studied in a more heterogeneous group of soccer players, larger effect sizes would likely have been found for at least some of these predictors [1, 33] (e.g. Franks et al. [106]).

Although the issue described above sounds straightforward, the effects of range restriction are often not explicitly taken into account in talent identification research. Range restriction is generally an issue when the aim of a study is to generalize results obtained from a specific selected group of elite players to a more general group, which is often the case when we study relationships between performance criterion variables and predictors. Aside from general issues such as insufficient power, careful consideration of the homogeneity of the participant group, in terms of the predictors the study examines, is also required to accurately interpret why certain relationships were or were not found. This is important because the ability of predictors to differentiate between players also depends on the degree of restriction in the sample. For example, some evidence suggests that a physiological sign such as sprinting ability is more suitable for differentiating between performance levels for relatively younger (e.g. U14–U16) than for older (e.g. U17–U19) skilled players [48, 73, 107], probably because the former group is more physically diverse, less exposed to systematic training, and not as strongly preselected on this variable. Some talent identification researchers relate their findings to the homogeneity of the sample and acknowledge that the discriminating or predictive value likely changes with the competitive level [48, 56, 73]. However, findings to date have been too inconsistent across studies to accurately determine what is important for any specific age group or skill level.

Thus, restriction of range is common in talent identification research, but is rarely considered explicitly when the generalizability of predictive validities is discussed (see Table 1).

### The Base Rate and the Utility of Talent Identification Programs

Successful talent identification procedures strive to select individuals who will attain excellent performance, and reject individuals who will not [22]. The focus of talent identification research is on the predictive value of different performance indicators; however, the practical usefulness or *utility* of these predictors, in terms of correctly identified players, is often not considered when evaluating the effectiveness of talent identification programs [32, 33].

The utility of selection procedures is greatly affected by contextual factors, especially the base rate and the selection ratio. The base rate is the proportion of individuals in the population of interest who are able to reach satisfactory criterion performance, that is, the proportion of individuals performing successfully if there is *no* selection [108]. Thus, the base rate is the prior probability of success for any given candidate [109]. Naturally, the base rate depends on the population of interest (i.e. the candidate pool) and on the criterion of interest. For example, several prospective cohort studies aimed to predict elite adult or late adolescent soccer success on the basis of performance indicators in groups of early adolescent players who were selected from large populations [43, 45]. This context is characterized by a very low base rate because very few young players have the ability to attain the elite adult level [110]. The base rate is higher when we consider, for example, strongly preselected older players in an elite youth academy, and when our criterion is operationalized as progressing to next year’s age class in the academy [52, 57, 107].

The selection ratio is defined as the proportion of players in the population of interest that is selected [108]. The selection ratio and the base rate are easily confounded in the soccer talent identification literature because the selection decision is often used as the criterion measure in this research field, as discussed in Sect. 2.1. Yet, they are essentially different and need to be defined separately in order to estimate the utility of a predictor.

The base rate, the selection ratio, and an unrestricted correlation coefficient between the predictor and the criterion can be used in utility models to estimate the gain in criterion performance as a result of using a particular predictor [30, 33]. There are several utility models, mostly developed in the context of personnel selection [108, 111–113]. As an example, we provide a description of the simplest model, the Taylor and Russell model [108].

**Fig. 1:**
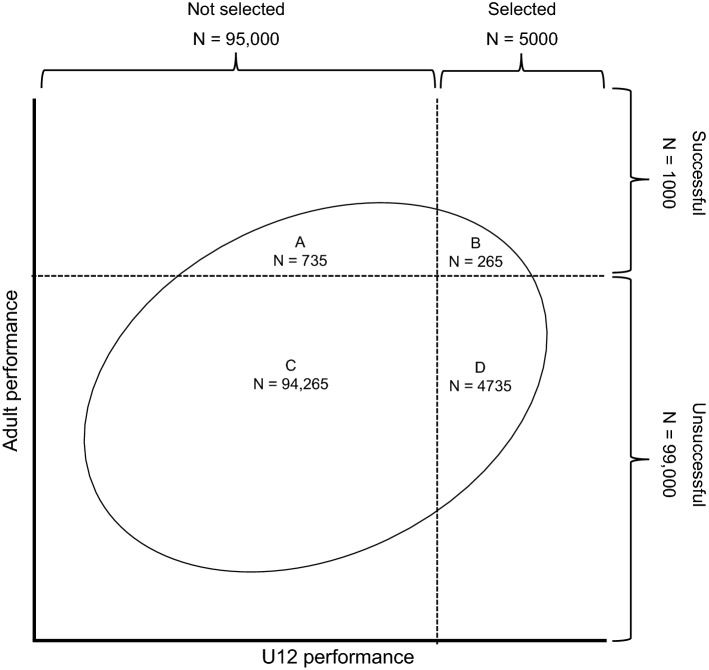
Visual representation of the example regarding the selection procedure of talented U12 players (*N* = 100,000). *A* = wrongfully rejected (false negatives); *B* = rightfully accepted; *C* = rightfully rejected; *D* = wrongfully accepted (false positives). *B*/(*B* + *D*) = sensitivity, whereas *C*/(*C* + *A*) = specificity Adapted from Taylor and Russell [108], with permission

In the Taylor and Russell model, a continuous criterion variable is dichotomized into a ‘successful’ and ‘unsuccessful’ group, based on a certain cut-off value used to define successful performance. Subsequently, utility is defined as the proportional increase in successful soccer players among those who are selected (the success ratio), resulting from using a specific selection procedure, compared with having no selection procedure (the base rate), or compared with the success ratio that would result from using a different selection procedure. In selection decisions, four groups can thus be distinguished: selected athletes who are successful (true positives), selected athletes who are unsuccessful (false positives), unselected athletes who would have been successful (false negatives), and unselected athletes who would not have been successful (true negatives). Accordingly, the proportion of true positives among all selected candidates corresponds to the sensitivity of a selection procedure, whereas the proportion of true negatives among all unselected candidates corresponds to the specificity. These terms are often used in medical research. Figure 1 visually represents these areas. In general, procedures with a high predictive validity, applied in contexts with a low selection ratio and a base rate that yields balanced groups of ‘suitable’ and ‘unsuitable’ players (approximately 0.50), yield the highest utilities. In addition, even when an assessment procedure has high predictive validity, utility will be relatively low when the selection ratio is high, and/or when the base rate is either very high or very low [108, 109].

Consider the following example. Assume that approximately 5000 U12 competence center players are selected annually from a total of 100,000 amateur club players (e.g. Höner and Votteler [43]), resulting in a selection ratio of 5%. Furthermore, they are selected based on a procedure that shows an unrestricted correlation of *r *= 0.4 with elite adult soccer performance. Note that *r *= 0.4 suggests relatively high predictive validity, especially considering the complexity in predicting a performance outcome of young players several years in the future from the time of testing [33]. In addition, only 1% of the population of U12 players (i.e. 1000 players) has the ability to obtain excellent elite adult soccer performance (the base rate). With this information, the success ratio resulting from the talent identification procedure can be computed (e.g. by using an online Theoretical Expectancy Calculator [114]).

The results based on this example are shown in Fig. 1. We obtained a success ratio of 5.3%, which means that only 5.3% (265/5000) of the selected players will be successful in achieving elite adult soccer performance. This may seem like a modest result; however, compared with the base rate of 1%, this may be a substantial increase. Moreover, 73.5% (735/1000) of all ‘suitable’ players among the population of U12 players are not selected. Conversely, of the 99,000 players who do not have the ability to be successful, approximately 95% (94,265/99,000) are not selected.

This example demonstrates how the base rate and the selection ratio can influence expectations regarding the utility of talent identification procedures for performance predictions [32]. To date, the talent identification literature has not generally taken this into account. We were able to identify one study within the talent identification literature that considered utility [43], whereas the effect of the base rate on the usefulness of the examined predictors was not discussed in the other studies in Table 1.

## Discussion and Suggestions for Future Research

The aim of this position paper was to evaluate the methodology in the soccer talent identification literature based on common principles from selection psychology that are relevant for talent identification research. We are aware that talent identification, in particular at younger ages, is very difficult [10, 32], yet we also believe that selection in general can provide players with realistic opportunities for successful development, and is often necessary from a practical point of view [115]. An important challenge therefore is to develop best-practice selection methods with clearly established predictive validity and reliability. The realization of a coherent body of knowledge regarding the prediction of soccer performance should ultimately provide guidelines for stakeholders and practitioners in talent identification. Considering the four topics discussed in this paper, we suggest that future talent identification studies in soccer consider the following points in order to help advance research practices and increase their practical and scientific impact.

### Develop Criterion Measures of Individual Soccer Performance

First, we suggest that future studies pay more attention to the criterion variables used in talent identification research, and develop individual soccer performance measures. More specifically, future studies may develop criterion measures that are not essentially selection decisions, and that can describe individual differences within selected groups of players to investigate what characteristics are related to which kind of soccer performance.

It should be emphasized that the development of such methods is a complicated task because of the dynamic nature of soccer. Elite individual soccer performance emerges through the complex interactions between the person and environmental constraints [60, 103]. As of yet, there is simply no single, objective measure of soccer performance available that can capture these complex interactions. Individual performance is dependent on the abilities of both teammates and opponents, which makes valid and reliable measurements very challenging [116]. The comparison of individuals’ soccer performance is complicated even further when we consider that different positions require different tasks and skills [58].

Despite the challenges, we believe that efforts to devise meaningful criterion measures are necessary to clearly establish predictor–criterion relationships. The literature is limited in providing measures that can describe individual performance differences, keep the person–task–environment relation intact, and account for the complex interactions between teammates and opponents [117]. Yet, there are several ways to obtain individual soccer performance measures that may provide a useful step in the right direction. For example, notation data on the frequency and quality of match events (e.g. Waldron and Worsfold [40], van Maarseveen et al. [118]) may be weighted and combined to assess performance per position. The weights of the events that are relevant for different positions can be determined by experts, such as coaches or scouts, or through machine-learning approaches when large amounts of data are available [72]. Furthermore, positional data (e.g. Frencken et al. [119], Memmert et al. [120]) may be used to quantify spatial-temporal patterns of play, which may be related to individual in-game success. Both these tools can be used to construct composite measures of ‘general’ soccer performance [72], or to measure a specific aspect of performance, such as passing [121], when the emphasis is on assessing the tasks of a specific player position [31]. Finally, simpler measures such as structured expert ratings are efficient tools for quantitatively evaluating individual performance [122], but it should be kept in mind that these also introduce more subjectivity, which can lead to biases and low interrater reliability [123]. Most importantly, studies are warranted that evaluate the validity and reliability of criterion measures, before they are implemented in predictive talent identification research.

### Close the Gap between Predictor and Criterion Variables

Second, we suggest that future studies explore the use of predictors that are more in line with the criterion. Specifically, talent identification research may broaden its current focus on low-fidelity signs as predictors to include high-fidelity samples as predictors of performance. With respect to the notion of behavioral consistency, several recent studies have demonstrated that prior competitive success in different sports is a relatively good predictor of short-term (i.e. 1–2 years) success [10, 124–126]. However, studies on soccer generally based individual performance on the highest (inter)national level of competition reached, which is less relevant for soccer talent identification procedures, and also suffers from limitations regarding the categorization of players. Therefore, it will be interesting to see whether samples of past soccer performance as predictors yield higher predictive validities of future individual soccer performance, compared with signs.

Match event data, positional data, and structured ratings can also be used to develop predictors by quantifying performance in sample-based assessment procedures, such as SSGs or 11-a-side games. However, it is important to note that similar to using an individual soccer criterion measure, measurements based on sample-based predictors may pose challenges related to the complex nature of soccer performance, including the dependence of individual performance on teammates and opponents, comparing different positions and competitions, and biases related to judgment. The reliability of such measurements needs to be investigated in future studies to develop optimally valid measures. Accordingly, recent efforts have been made to develop reliable structured rating forms to measure performance in SSGs [118, 127]. As mentioned by other researchers [1, 8, 22, 128], performance should preferably be assessed longitudinally over a series of games in order to obtain reliable assessments of individual soccer performance based on these samples. In addition, when a researcher aims to investigate match performance for a given group of players, and has control over the organization of the games, the performance level of opponents and teammates can be controlled for by reorganizing players into different teams after each (small-sided) game, as was done by Fenner et al. [69].

### Consider Restriction of Range

Third, future studies should take into account the potential effect of range restriction on their conclusions by carefully considering the homogeneity of their study participants in terms of physical, physiological, and other soccer-related characteristics. Subsequently, researchers should clearly state the population to which findings may be generalized. In strongly restricted samples, the absence of observed predictor–criterion relationships does not necessarily imply that a predictor is not positively related to attaining elite performance in the general population, or to the initial performance level prior to the selection decision. In addition, which predictors are useful for differentiating between players probably depends on the level of expertise, and hence the degree of preselection, in the population of interest. Future research could pay close attention to which predictors work in which specific populations.

It should be noted that correcting for the effects of range restriction has been challenging in talent identification research. Range restriction is an issue that occurs in most selection contexts, including personnel and educational selection. In a typical selection study, the entire candidate pool would be assessed on the predictor variables, but criterion performance data are only available for the candidates who were selected. The resulting underestimated predictor–criterion relationship can be corrected using several available formulas [105, 129], which yield estimates of the predictor–criterion relationship in the unrestricted population of interest [105, 130]. These corrections are often applied in the selection psychology literature [131]. However, they have not been used in a talent identification context, which is most likely due to the design of most talent identification studies; because performance level or a selection decision functions as the criterion, range restriction does not occur *within* the sample(s) under study. Accordingly, when the design of future studies includes soccer criterion measures that can differentiate between individual players’ performance after selection, range-restricted relationships can be accounted and corrected for using correction formulas that take the variance in the candidate pool into account [105, 130].

### Identify the Utility of Predictors

Finally, we suggest that future studies discuss the potential utility of predictors more often, and consider realistic estimates of contextual factors such as the base rate and the selection ratio. For instance, future studies may investigate how novel predictors compare with current selection decisions made by coaches and scouts, in terms of incremental validity and utility. We acknowledge that it is difficult to obtain estimates of the base rate based on empirical data. However, an educated guess about a range of plausible values of the base rate [132] can be obtained based on interactions with experts, such as by asking several coaches or scouts to estimate the proportion of players who they think have the potential to obtain excellence. That range of plausible values can be used in utility models. Since this base rate is generally very low in talent identification contexts [33, 43], and arguably often lower than the selection ratio, not all selected players can become successful, regardless of the predictor’s validity. Therefore, we believe that utility estimates will help to create realistic expectations for researchers and stakeholders about talent identification procedures.

## Conclusion

In the current position paper we discussed several methodological issues common in the soccer talent identification literature, and provided suggestions to improve the methodological quality and robustness of research practices in future talent identification studies. We hope that the general principles discussed here will also transfer to practical selection contexts, and we believe that researchers have an important responsibility to communicate the reliability and validity of talent identification procedures to the sports field [133]. Thinking critically about the methodology and design of studies in sports opens the door for innovative research that advances this exciting field, and hopefully leads to a more coherent scientific and practical framework for talent identification.

## Footnotes

1 We chose to focus our discussion on the domain of soccer because most published studies on talent identification are focused on this sport, and talent identification procedures across sports are difficult to compare [15, 20]. However, our discussion can also be translated to other specific domains of open-skilled sports.
